# Supplementary material for: Modeling glioblastoma heterogeneity as a dynamic network of cell states
Source: Mol Syst Biol. 2021 Sep 16;17(9):e10105. doi: 10.15252/msb.202010105 (PMC8444284; doi:10.15252/msb.202010105)
Supplement: Supplementary file 6 — Source Data for Figure 5 [file MSB-17-e10105-s004.zip › Figure5A_sourcedata/GSEA_3071/hallmarks_stateI.GseaPreranked.1623742531165/HALLMARK_ESTROGEN_RESPONSE_LATE.html]

Details for gene set HALLMARK\_ESTROGEN\_RESPONSE\_LATE[GSEA]

|  || Dataset | state13071 |
| Phenotype | NoPhenotypeAvailable |
| Upregulated in class | na\_neg |
| GeneSet | HALLMARK\_ESTROGEN\_RESPONSE\_LATE |
| Enrichment Score (ES) | -0.26906598 |
| Normalized Enrichment Score (NES) | -1.3429078 |
| Nominal p-value | 0.1002994 |
| FDR q-value | 0.23446693 |
| FWER p-Value | 0.974 |
Table: GSEA Results Summary

  

Fig 1: Enrichment plot: HALLMARK\_ESTROGEN\_RESPONSE\_LATE      
 Profile of the Running ES Score & Positions of GeneSet Members on the Rank Ordered List

  

| PROBE | GENE SYMBOL | GENE\_TITLE | RANK IN GENE LIST | RANK METRIC SCORE | RUNNING ES | CORE ENRICHMENT || 1 | TFF3 |  |  | 18 | 1.667 | 0.0288 | No |
| 2 | SLC29A1 |  |  | 50 | 1.357 | 0.0475 | No |
| 3 | TPBG |  |  | 51 | 1.346 | 0.0749 | No |
| 4 | SLC9A3R1 |  |  | 57 | 1.322 | 0.1005 | No |
| 5 | SLC26A2 |  |  | 121 | 1.012 | 0.1028 | No |
| 6 | XBP1 |  |  | 215 | 0.797 | 0.0920 | No |
| 7 | UGDH |  |  | 238 | 0.759 | 0.1011 | No |
| 8 | DNAJC12 |  |  | 252 | 0.732 | 0.1123 | No |
| 9 | IGFBP4 |  |  | 262 | 0.725 | 0.1244 | No |
| 10 | PRSS23 |  |  | 264 | 0.721 | 0.1389 | No |
| 11 | PDCD4 |  |  | 295 | 0.682 | 0.1441 | No |
| 12 | MYOF |  |  | 310 | 0.663 | 0.1535 | No |
| 13 | ATP2B4 |  |  | 330 | 0.644 | 0.1611 | No |
| 14 | TIAM1 |  |  | 341 | 0.636 | 0.1712 | No |
| 15 | BLVRB |  |  | 535 | 0.488 | 0.1250 | No |
| 16 | PDLIM3 |  |  | 547 | 0.481 | 0.1316 | No |
| 17 | CPE |  |  | 680 | 0.420 | 0.1018 | No |
| 18 | KLF4 |  |  | 742 | 0.400 | 0.0922 | No |
| 19 | RPS6KA2 |  |  | 817 | 0.376 | 0.0783 | No |
| 20 | CLIC3 |  |  | 821 | 0.375 | 0.0851 | No |
| 21 | FOS |  |  | 832 | 0.370 | 0.0897 | No |
| 22 | OPN3 |  |  | 839 | 0.369 | 0.0955 | No |
| 23 | JAK2 |  |  | 886 | 0.353 | 0.0893 | No |
| 24 | PAPSS2 |  |  | 898 | 0.351 | 0.0933 | No |
| 25 | DYNLT3 |  |  | 946 | 0.340 | 0.0866 | No |
| 26 | SLC7A5 |  |  | 1084 | 0.310 | 0.0530 | No |
| 27 | HR |  |  | 1101 | 0.306 | 0.0546 | No |
| 28 | JAK1 |  |  | 1145 | 0.299 | 0.0482 | No |
| 29 | SLC1A4 |  |  | 1184 | 0.292 | 0.0431 | No |
| 30 | IL6ST |  |  | 1215 | 0.285 | 0.0402 | No |
| 31 | ADD3 |  |  | 1261 | 0.275 | 0.0327 | No |
| 32 | IDH2 |  |  | 1324 | 0.264 | 0.0201 | No |
| 33 | CAV1 |  |  | 1341 | 0.261 | 0.0207 | No |
| 34 | CD44 |  |  | 1430 | -0.251 | 0.0002 | No |
| 35 | FARP1 |  |  | 1496 | -0.257 | -0.0135 | No |
| 36 | FLNB |  |  | 1591 | -0.267 | -0.0354 | No |
| 37 | GALE |  |  | 1592 | -0.267 | -0.0299 | No |
| 38 | MAPT |  |  | 1627 | -0.270 | -0.0343 | No |
| 39 | BAG1 |  |  | 1630 | -0.270 | -0.0294 | No |
| 40 | NXT1 |  |  | 1722 | -0.283 | -0.0501 | No |
| 41 | TST |  |  | 1779 | -0.291 | -0.0604 | No |
| 42 | ITPK1 |  |  | 2019 | -0.320 | -0.1235 | No |
| 43 | ALDH3A2 |  |  | 2040 | -0.324 | -0.1227 | No |
| 44 | NRIP1 |  |  | 2069 | -0.329 | -0.1241 | No |
| 45 | HSPA4L |  |  | 2105 | -0.335 | -0.1275 | No |
| 46 | FDFT1 |  |  | 2117 | -0.337 | -0.1238 | No |
| 47 | DCXR |  |  | 2157 | -0.344 | -0.1281 | No |
| 48 | NMU |  |  | 2166 | -0.346 | -0.1234 | No |
| 49 | PLK4 |  |  | 2167 | -0.346 | -0.1163 | No |
| 50 | SCARB1 |  |  | 2273 | -0.366 | -0.1394 | No |
| 51 | STIL |  |  | 2374 | -0.388 | -0.1606 | No |
| 52 | CD9 |  |  | 2444 | -0.400 | -0.1725 | No |
| 53 | HPRT1 |  |  | 2454 | -0.402 | -0.1669 | No |
| 54 | CDC20 |  |  | 2540 | -0.425 | -0.1830 | No |
| 55 | PPIF |  |  | 2624 | -0.446 | -0.1981 | No |
| 56 | COX6C |  |  | 2825 | -0.511 | -0.2458 | No |
| 57 | TOP2A |  |  | 2842 | -0.517 | -0.2400 | No |
| 58 | FKBP4 |  |  | 2871 | -0.528 | -0.2373 | No |
| 59 | OLFM1 |  |  | 2981 | -0.589 | -0.2570 | Yes |
| 60 | CELSR2 |  |  | 2993 | -0.597 | -0.2481 | Yes |
| 61 | GAL |  |  | 3046 | -0.631 | -0.2503 | Yes |
| 62 | RBBP8 |  |  | 3053 | -0.637 | -0.2390 | Yes |
| 63 | CCND1 |  |  | 3072 | -0.655 | -0.2309 | Yes |
| 64 | HOMER2 |  |  | 3087 | -0.665 | -0.2214 | Yes |
| 65 | BTG3 |  |  | 3148 | -0.727 | -0.2241 | Yes |
| 66 | CDC6 |  |  | 3204 | -0.785 | -0.2240 | Yes |
| 67 | MDK |  |  | 3224 | -0.811 | -0.2130 | Yes |
| 68 | MEST |  |  | 3307 | -0.940 | -0.2177 | Yes |
| 69 | EMP2 |  |  | 3338 | -0.998 | -0.2060 | Yes |
| 70 | ID2 |  |  | 3374 | -1.110 | -0.1936 | Yes |
| 71 | GINS2 |  |  | 3386 | -1.156 | -0.1731 | Yes |
| 72 | NBL1 |  |  | 3432 | -1.368 | -0.1583 | Yes |
| 73 | CHPT1 |  |  | 3434 | -1.385 | -0.1303 | Yes |
| 74 | FABP5 |  |  | 3437 | -1.400 | -0.1023 | Yes |
| 75 | CXCL14 |  |  | 3461 | -1.710 | -0.0741 | Yes |
| 76 | ASCL1 |  |  | 3484 | -1.937 | -0.0410 | Yes |
| 77 | CKB |  |  | 3497 | -2.376 | 0.0041 | Yes |
Table: GSEA details [plain text format]

  

Fig 2: HALLMARK\_ESTROGEN\_RESPONSE\_LATE: Random ES distribution      
 Gene set null distribution of ES for **HALLMARK\_ESTROGEN\_RESPONSE\_LATE**
